# Predictive factors for outcome in adolescents with anorexia nervosa: To what extent does parental Expressed Emotion play a role?

**DOI:** 10.1371/journal.pone.0196820

**Published:** 2018-07-31

**Authors:** Jeanne Duclos, Géraldine Dorard, Solange Cook-Darzens, Florence Curt, Sophie Faucher, Sylvie Berthoz, Bruno Falissard, Nathalie Godart

**Affiliations:** 1 Psychiatry Unit, Institut Mutualiste Montsouris, Paris, France; 2 CESP, INSERM, UMR 1018, University Paris-Sud, UVSQ, Université Paris-Saclay, Paris, France; 3 Laboratoire de Psychopathologie et Processus de santé (LPPS), University Paris Descartes, Boulogne, France; 4 Service de psychopathologie de l’enfant et de l’adolescent, Hôpital Robert Debré, Paris, France; 5 Fondation de Santé des Etudiants de France, Paris, France; Hospital Universitari de Bellvitge, SPAIN

## Abstract

In studies on family therapy in Anorexia Nervosa, family relationships, as assessed by Expressed Emotion, have been associated with outcome. Our aim was to explore the contribution of Expressed Emotion as a predictor of 18-month outcome, above and beyond the usual predictive factors. Sixty adolescent girls suffering from Anorexia Nervosa and their parents were assessed at baseline and 18 months later. Levels of Expressed Emotion were evaluated in both parents with the Five-Minute Speech Sample. After controlling for treatment group and initial clinical status, high maternal Emotional Over-Involvement at baseline was significantly associated with better clinical state. More precisely, high maternal Emotional Over-Involvement was associated with higher nutritional status, lower eating disorder severity and fewer re-hospitalizations 18 months later. No associations were found with paternal levels of Expressed Emotion. Therefore, our study confirmed the importance of taking into account both maternal and paternal Expressed Emotion. Our results also underlined that high maternal Emotional Over-Involvement plays a positive role in the outcome of Anorexia Nervosa and needs to be explored further.

## Introduction

Since Minuchin’s work [1], family therapy (FT) has become the gold standard treatment for adolescents with anorexia nervosa (AN). The essential role of the family in its treatment has been approved and accredited by the whole AN community [2, 3]. Indeed, family-based treatment (FBT) and systemic FT have demonstrated some efficacy in randomized-controlled trials (RCT) conducted on AN adolescents [4–8]. However, less than half the patients recover using this approach [8]. It is therefore crucial to more fully understand the predictive factors of therapeutic outcome. Among these factors, family dimensions, and more specifically Expressed Emotion (EE), have been implicated in the field of eating disorders (ED) [9, 10]. EE was originally obtained in the context of patients with schizophrenia [11, 12] who were primarily rated on the basis of a semi-structured interview known as the Camberwell Family Interview (CFI) [10–13]. The CFI rated parent-child interactions, including critical comments, positive remarks, hostility, warmth and emotional over-involvement (EOI). Because the CFI is particularly timeconsuming, shorter and more cost effective instruments were developed, such as the Five-Minute Speech Sample (FMSS) [14–15]. Although the predictive validity of the FMSS has at times been questioned in schizophrenia in comparison with the CFI, more recent publications have confirmed its validity, particularly in the field of eating disorders [10, 11, 15–17]. The FMSS involves two EE components: Critical and EOI [10, 14–15]. Criticism captures blame, dislike or resentment that parents may feel toward their child. EOI includes overprotection and self-sacrificing, lack of objectivity, excessive detail about the past, emotional displays (e.g., crying), as well as statements of attitude (i.e., extreme loving and willingness to do anything for the child in the future) and more than five positive remarks regarding the child [10, 13–19]. Until very recently, EE components have been a negative force, even though recent research focused on its positive aspect. Indeed, FMSS-EOI includes both positive and negative components [15, 16].

Thus, EE is a bi-directional concept which involves a non-direct causal link focusing on both positive and negative parental patterns of interaction with their child suffering from mental illness into day-to-day family life [11–13, 15, 18–22].

More importantly, EE level is a relevant predictor of treatment compliance, early treatment outcome and long-term clinical outcomes in AN. Indeed, a high level of maternal criticism has been associated with a poorer outcome [20, 21, 23–26] while parental warmth has been found to be a predictor of good outcome [21,27]. Furthermore, patients from low EE families fare better in treatment than patients belonging to high EE families [20]. Parental EE levels are usually observed to decrease during the course of treatment [9, 10, 20–22, 28]. Findings suggest that greater emphasis on parent support during treatment may improve outcomes [9, 10, 27, 28]. Recent studies found that EE is mediated by parental characteristics and patient illness-related characteristics with a parental gender effect [18–21]. Maternal Critical and EOI EE were related both to the severity of the daughters’ clinical state and to higher maternal psychological distress [18, 19]. While paternal EOI was related to their own levels of anxiety [18] and was significantly associated with more patient eating disorder symptoms [10]. It agrees with our separate assessment of paternal and maternal EE levels [18, 19].

In addition to EE, other predictive factors (i.e. number of previous treatments, duration of illness) seem to play a role in outcome [23, 24]; but to date no family therapy RCTs among adolescents with AN have investigated family and other predictive factors conjointly [5].

To summarize, research has traditionally focused on the negative aspects of EE and positive aspects of EOI and their association with a better outcome are novel in ED research [15, 20–22, 27]. And, due to the dramatic predictive power and relative low cost of FMSS-EE, this tool has been widely employed in EE research [15].

The purpose of this study was to investigate the extent to which family relationships, evaluated by FMSS-EE, remained a significant predictive factor of outcome above and beyond other commonly explored predictive factors.

## Materials and methods

### Procedures and ethics

This study was part of a RCT on the efficacy of systemic FT (see [6] for a detailed description of the design). The Trial Registration is Controlled-trials.com ISRCTN71142875. This study received approval from the Ile-de-France III Ethics Committee and was in accordance with the terms of the Helsinki declaration.

Prior to inclusion in the study, all participants were hospitalized on our French inpatient care unit for life-threatening physical and/or mental conditions related to AN (including a body mass index (BMI) below 14 and/or rapid weight loss and/or compromised vital functions, severe depression, high suicidal risk, chronic under-nutrition with low weight, and/or failure of outpatient care).

Once the patient was admitted, the objectives of inpatient care were defined by means of a weight contract establishing a discharge target weight [6, 29–31]. Although each patient and her parents were informed of the study at admission, inclusion and randomization occurred in the second half of their hospital stay (i.e. half-way towards their target weight), a period during which the post-hospitalisation programme is being defined.

At the time of the evaluation, patients were hospitalized and were in regular contact with their parents: indeed, they spent the weekend at home and had two 2-hour visits during the week on average. Patients had been living with their parents before inpatient treatment.

*Inclusion criteria*: female, 13–21 years of age, DSM-IV criteria for AN [32], aged ≤ 19 years at AN onset, illness duration ≤ 3 years, living within commuting distance of the study site, and never received family therapy.

*Exclusion criteria for both patients and their parents were*: an insufficient command of the French language; for patients, any potentially confounding metabolic pathology (e.g. diabetes) or psychotic disorder.

### Participants

A total of 60 families were included between January 1999 and July 2002.

At baseline, the mean age of the 60 patients was 16.6 years (SD = 1.6). The mean age at onset of AN was 14.8 years (1.6) and the mean duration of illness was 16.6 months (6.8). Eighty-seven percent of the patients were suffering from AN restrictive subtype (n = 52). The mean minimum lifetime BMI was 13.0 (1.1). The mean duration of hospitalization was 21 weeks (13.9). The number of previous hospitalizations varied from 0 to 4, and a quarter of the patients had previously been hospitalized once. All patients had amenorrhea and a mean BMI at discharge over the 10th percentile (i.e. 17 kg/m2 for 16.5 to 16.9 year-old). They were all living with theirs parents before hospitalisation.

The mean age of the 55 fathers was 49.4 (4.9) years and that of the 58 mothers was 47.6 (4.8). According to French classifications, 74.1% of the fathers and 82.3% of the mothers had high socio-economic status. Concerning the family status, 85% (N = 51) of our sample lived in intact families.

### Outpatient treatment program

Two treatments were used in the RCT: Treatment-As-Usual (TAU) and TAU+ systemic FT that were administered on a post-hospitalization outpatient basis over a period of 18 months [6, 7].

*TAU* involves ambulatory care which is organized before hospital discharge and is tailored according to the mental and physical state of the patient [29, 30]. It includes individual consultations, regular interviews involving the parents, and, if required, individual psychotherapy with another therapist. The sessions last approximately 30 minutes and take place every three to four weeks.

*Systemic FT* is designed by our team as one component of a multi-dimensional outpatient care program [29, 33]. Sessions focus on familial dynamics as a whole and do not address eating behaviors directly (the latter being addressed by the coordinating psychiatrist). The sessions include the patient, the parents, and siblings if they are over the age of 6 and living in the home. They last approximately 1h30 and take place every three to four weeks.

It should be noted that neither treatment specifically addressed parental EE.

### Assessment

Participants completed several self-report questionnaires (including ad hoc sociodemographic and clinical data collection) plus investigator-based measures, at baseline (upon discharge) and at 18-month follow-up [6]. With the exception of Morgan-Russell Global Outcome Assessment Scale (GOAS) all selected measures have been validated on a French population. References to articles describing the French validation of the tools and their psychometric properties are provided below for each of them.

#### AN patients

- The Morgan-Russell (GOAS) [34, 35] (investigates the central clinical features in AN through 5 areas of functioning: Nutritional status, Menstrual function, Mental state, Sexual adjustement and Socioeconomic status. A quantitative score (0–12) is obtained and the higher the score, the better the clinical state.

The Russell et al. [36] methodology was used and two outcome categories were defined. Good and Intermediate Morgan-Russell (MR) outcome: weight >10^th^ BMI percentile and regular menstruation or amenorrhea; poor MR outcome: weight <10^th^ BMI percentile and/or presence of bulimic symptoms.

- Clinical data at baseline and 18-month follow-up were collected with an ad hoc structured clinical interview: minimum lifetime BMI; current BMI; age at AN onset; AN duration (in months); menstruation; contraceptive use; number of previous hospitalizations; duration of hospitalization preceding inclusion; re-admission in the course of follow-up.

- Participants also completed the validated French version of the Eating Disorder Inventory (EDI) [37, 38], a self-report questionnaire that assesses core features of eating disorder (ED) psychopathology. It comprises 64 items divided into eight subscales: Drive for thinness, Bulimia and Body dissatisfaction. Perfectionism, Interpersonal distrust, Ineffectiveness, Maturity fear and Interoceptive awareness. Responses are scored on a 6-point Likert scale and recoded into a 4-point scale, with a “0” as- signed to the three least symptomatic responses and a “3” assigned the most symptomatic responses. The Cronbach α (alpha) reliability index were as follows: drive for thinness 0.85; bulimia 0.90; body dissatisfaction 0.90; ineffectiveness 0.91; perfectionism 0.83; interpersonal distrust 0.82; interoceptive awareness 0.85; maturity fears 0.88. The Cronbach α reliability index for the entire score was 0.95. The score on each subscale and the total score are calculated [6, 39] was used in order to have a total score. EDI total score is obtained by the mean score from the eight subscales. Higher scores indicate more severe ED psychopathology.

#### Parents

- Sociodemographic data, including age and socioeconomic status of both parents, were collected.

- The validated French version of the Five-Minute Speech Sample (FMSS) [14, 16, 40] was administered to each parent and recorded to assess high or low levels on two EE dimensions: Critical EE and EOI EE. Each parent was asked to talk about what kind of person they thought their daughter was and their relationship with her.

Critical EE category is rated on the basis of the parent’s Initial Statement concerning the patient, the Relationship, Critical Comments and Dissatisfaction. High Critical EE is rated if the parent makes an initial negative statement and/or expresses a negative relationship and/or makes one or more Critical Comments; in other cases, Critical EE is rated as "Low".

EOI EE category is based on Emotional Display (e.g. the parent bursts into tears), Statements of (loving) Attitude (scored as present when the parent expresses very strong feelings of love for the relative or willingness to do anything for the relative in the future), Self-sacrifice / Overprotective behavior or Lack of objectivity (SOL: scored present when the parent believes the patient is always right, makes excuses for and/or rationalizes the patient’s behavior), Excessive Details about the past (rated present when the parent gives an inordinate amount of extraneous information about the child’s distant past) and Positive Remarks. A "High" EOI EE rating is based on self-sacrificing or overprotective attitudes and/or emotional display during the interview and on the presence of any 2 of the following: excessive details, statements of loving attitudes, at least 5 positive remarks; in other cases, EOI EE is rated as "Low".

As indicated in the introduction, the FMSS-EOI includes both positive and negative elements. The positive elements are the following “statements of loving attitudes”, “positive remarks”, while the negative dimensions are: “self-sacrifice / overprotective behavior” and "emotional display" [16, 40].

Assesment and coding of EE categories were conducted by two trained assessors (NG and Z. Rein) not involved in treatment delivery. Inter-rater agreement was calculated for both Critical EE (k_Crit_ = 0.810, p<0.05) and EOI (k_EOI_ = 0.785, p<0.05) with a final reconciliation [10, 16].

### Determination of the predictive factors

We examined a set of clinically relevant variables derived from the empirical litterature as potentially predictive factors of outcome, to characterize the patients’ clinical condition at baseline.

#### AN-related data

1) minimum lifetime BMI (kg/m2); 2) current BMI; 3) eating disorder psychopathology (EDI); 4) age at AN onset; 5) AN duration (in months); 6) duration of the index hospitalization (just preceding inclusion); 7) number of previous hospitalizations [23, 24, 41, 42].

#### Family relationships via EE

1) Critical EE; 2) EOI EE [9, 10, 21, 22].

### Statistical analysis

The completion of missing follow-up data was performed using the "last observation carried forward" procedure, which enabled the inclusion of 59 participants (29 TAU; 30 TAU + systemic FT) (see [6] for more details).

At 18 months, in case of contraceptive use, subjects with a BMI<10^th^ percentile were conservatively rated as presenting amenorrhea (n = 8) (as in previous studies [6, 43]).

In addition to EE main categories (high/low Critical and EOI EE), FMSS sub-dimensions were used when at least 5 observations per covariate were rated.

Since therapeutic effectiveness of the treatments (TAU and TAU+ systemic FT) was investigated in a previous article [6], the present research does not compare treatment conditions but integrated them as co-variables in all statistical analyses.

Descriptive statistics for quantitative measures (mean, variance, standard deviation) and for qualitative measures (percentage) were first calculated. The Chi^2^ or Fisher Exact Probability tests were used as appropriate for the categorical variables. Either Student t-tests or Mann-Whitney tests were used (as appropriate) for the continuous variables. The study of relationships between continuous variables was performed using Spearman’s correlation coefficient.

**Fig 1:**
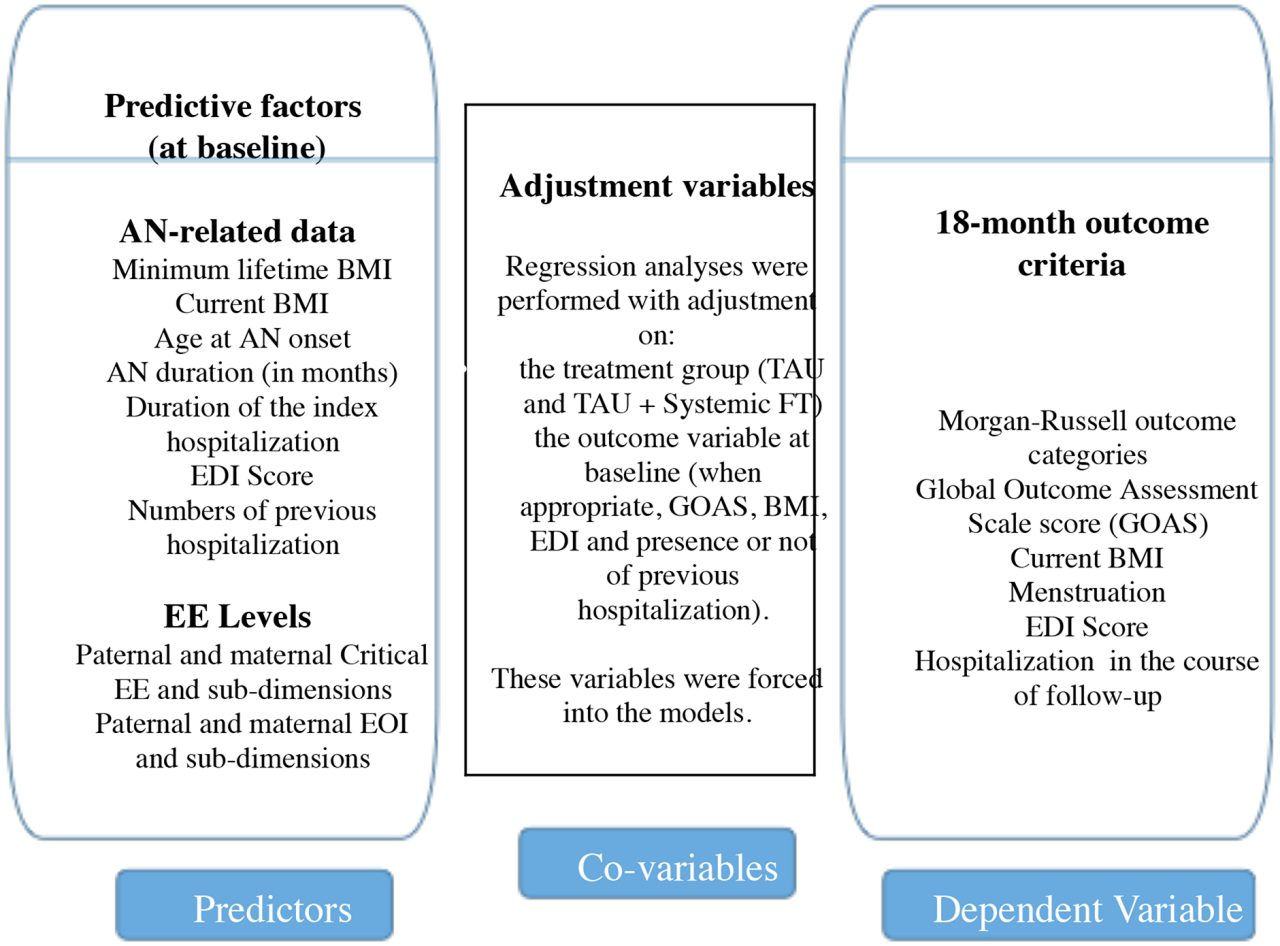
Statistical analysis: Predictive factors at baseline of the 18-month outcome criteria and adjustment variables. EE: Expressed Emotion; TAU: Treatment as Usual; systemic FT: Family Therapy; EOI: Emotional Over-Involvement; BMI: Body Mass Index; AN: Anorexia Nervosa.

Then, univariate linear regressions (for continuous variables) and univarite logistic regressions (for categorical variables) were calculated with AN-related data and EE levels at baseline as predictive variables, and 18-month outcome criteria as dependent variables (see Fig 1 for the list of variables).

Lastly, in order to determine which variables contributed the most to the 18-month outcome, multivariate linear and logistic regressions were calculated with AN-related data and EE levels at baseline that exhibited a significant link in the univariate analysis.

The effect size was evaluated for qualitative variables by the odds ratio (OR) with a 95% confidence interval (CI) as recommended by Fleiss [44] and for quantitative variables by Cohen’s d test. Since our research was not designed to lead to a single final conclusion, comparisons were not adjusted for multiple testing (i.e. Bonferroni correction [45]).

All statistical analyses were performed with the IBM SPSS Statistics 20 software, using two-tailed statistical tests and a level of significance of .05.

## Results

### Evolution of the patients’ clinical characteristics between baseline and 18-month follow-up (Table 1)

**Table 1:** Evolution of essential clinical characteristics between baseline and 18-month follow-up.

	Baseline (N = 60)	18 month FU (N = 59)*	p-values	Relative Effect Size OR [95% CI]
GOAS, mean (SD)	4.3 (1.1)	7.4 (2.2)	.097	-1.80 [-2.8;-1.24]
BMI, mean (SD)	16.9 (1.1)	17.6 (2.2)	.012	-.41 [-.69;.15]
EDI, mean (SD)	60.7 (35.1)	47.8 (28.9)	<.01	.4 [-8.48;7.78]

FU: Follow-Up; GOAS: Morgan and Russell Global Outcome Assessment Schedule; SD: Standard Deviation; BMI: Body Mass Index; EDI: Eating Disorder Inventory;

*1 patient lost for follow-up; NS: Non-significant; 95% CI: 95% confidence interval.

The 60 patients showed significant improvement on AN-related data between baseline and 18-month follow-up; BMI and the EDI total score were also improved. Furthermore, 17 patients (28.8%) have a good or intermediate MR outcome and almost half of them (29 (49,1%)) did not have amenorrhea any longer.

### Level of parental Expressed Emotion (EE) (Table 2)

**Table 2:** Parental levels of Expressed Emotion at baseline.

Levels of EE (FMSS)	Fathers N (%)	Mothers N (%)
**High Critical EE**	**15 (27.3)**	**14 (24.1)**
Initial Statement (-)	4 (6.7)	2 (3.4)
Relationship (-)	3 (5.5)	4 (6.9)
Critical Comments (P)	11 (20)*	14 (24.1)*
Dissatisfaction (P)	21 (38.2)*	21 (36.2)*
**High EOI EE**	**19 (34.5)**	**21 (36.2)**
Emotional Display (P)	3 (5.5)	3 (5.2)
Statement of (loving) Attitudes (P)	8 (14.5)*	6 (10.3)*
SOL (P)	1 (1.8)	2 (3.4)
Excessive Detail about the Past (P)	21 (38.2)*	24 (41.4)*
Positive Remarks (P)	4 (7.2)	8 (13.8)*

EE: Expressed Emotion; EOI: Emotional Over-Involvement; FMSS: Five Minutes Speech Sample; N: sample size; P: present; (-): negative; SOL: Self-Sacrifice /Overprotective /Lack of Objectivity;

*: FMSS sub-dimensions that were used in analysis when at least 5 observations per covariate were coded

Fewer than 30% of the parents (the 55 mothers and 58 fathers alike) had high levels of Critical EE, while over 30% of them had high levels of EOI. Parental EOI EE was for the most part derived from Statements of (loving) Attitudes and Positive Remarks.

### Associations between 18-month outcome criteria and predictive factors at baseline

#### Univariate regression analyses

Revealed no significant association between age at AN onset, minimum lifetime BMI, AN duration, and number of previous hospitalizations on the one hand, and any of the considered outcome criteria on the other. No significant association was found between parental Critical EE and paternal EOI and any of the outcome criteria (S1 Table).

#### Multivariate linear and logistic regressions

Were then calculated with those AN-related data and EE levels at baseline that exhibited a significant link in the univariate analysis (Table 3).

**Table 3:** Multivariate regression models predictors of 18-month outcome criteria.

18-month Outcome Criteria	Factors at baseline predictive of outcome	R2	p-value	unstandardized β	Related effect size OR [95% CI]
**Good and Intermediate MR outcome categories**	**- Maternal Excessive Details about the Past**	**.269**	**.008**	**NA**	**.156 [.039; .616]**
	**- Treatment Group**		**.023**		**.194 [.047; .795]**
**GOAS score**	**- Maternal High EOI**	**.119**	**.056**	**1.200**	**.260 [-.031; 2.431]**
	**- GOAS at baseline**		**.196**	**.328**	**.172 [-.175; .832]**
	**- Treatment Group**		**.668**	**.252**	**.057 [-.920; 1.424]**
**BMI**	**- Maternal Excessive Details about the Past**	**.217**	**.010**	**1.507**	**.330 [.373; 2.640]**
	**- Maternal Positive relationship**		**.811**	**.114**	**.030 [-.834; 1.062]**
	**- BMI at baseline**		**.022**	**.623**	**.296 [.094; 1.151]**
	**- Treatment Group**		**.331**	**.548**	**.122 [-.572; 1.669]**
**Menstruation**	**- Paternal excessive details about the past**	**.222**	**.082**	**NA**	**.335**
	**- EDI**		**.181**		**1.012**
	**- Treatment Group**		**.093**		**2.748**
**EDI Score**	**- Maternal Statement of Loving Attitude**	**.539**	**.016**	**-23.054**	**-.255 [-41.539; -1.148]**
	**- Duration of index hospitalization**		**.622**	**.106**	**.052 [-.323; .535]**
	**- EDI at baseline**		**<.001**	**.515**	**.624 [.341; .690]**
	**- Treatment Group**		**.618**	**2.927**	**.051 [-8.806; 14.661]**
**Re-hospitalization for AN or other psychiatric disorder**	**- Maternal High EOI**	**.303**	**.007**	**-1.807**	**.164 [.044; .611]**
	**- Duration of index hospitalization**		**.036**	**.053**	**1.054 [1.003; 1.107]**
	**- Treatment Group**		**.298**	**-.641**	**.527 [.157; 1.763]**

EE: Expressed Emotion; MR: Morgan and Russell; Treatment Group: Treatment as Usual (TAU) and TAU + systemic Family Therapy; GOAS: Morgan and Russell Global Outcome Assessment Scale; EOI: Emotional Over-Involvement; BMI: Body Mass Index; EDI: Eating Disorder Inventory; AN: Anorexia Nervosa; 95% CI: 95% Confidence Interval; OR = Odds Ratio; R^2^ Nagelkerke; NA: non-applicable

Controlling for treatment group and initial clinical status, high maternal EOI was significantly associated with fewer re-hospitalizations and a trend was observed for better clinical status on the GOAS. Regarding EOI subscales, maternal Excessive Details about the Past was significantly associated with good and intermediate MR outcome categories and higher BMI. Maternal Statement of (Loving) Attitude was associated with lesser intensity of eating disorders 18 months later. The duration of the index hospitalization was associated with risk of re-admission for AN or other psychiatric disorders during the follow-up.

## Discussion

Our objective was to determine the extent to which family relationships, as assessed by EE, would be predictive of 18-month outcome for adolescent girls with AN once other commonly explored predictive factors were also included in the analyses.

One of the main strengths of our study is to consider maternal and paternal EE influences distinctly, beyond global parental EE [18, 20, 21]. Taking into account each parent’s influence is crucial in research [18, 19] as well as in clinical settings. Indeed, in most family work, clinicians target both family trajectories (i.e., couples) and dyadic interactions (i.e., father-daughter and mother-daughter). Hence, our results highlighted the need to consider the impact of dyadic relationships independently in addition to the parental couple.

Traditionally, EE studies have focused on the criticism dimension [22–26, 46]. Unlike these previous studies, our results did not evidence any impact of critical EE or critical comments by the parents on patient outcome. Only a few recent researches examined the positive aspects of EE [20, 21, 27].

Surprisingly, one of our key results was the considerable contribution of maternal EOI to better patient outcome. Van Furth et al. [47] showed that higher maternal EOI levels, using the Camberwell Family Interview (CFI), were linked to poorer outcome one year after treatment in 49 adolescents with eating disorders. These conflicting results could be explained in terms of the choice of EE measurement. In the CFI, EOI has an exclusively negative connotation while the FMSS-EOI includes both positive and negative elements [16, 40]. In our population, maternal EOI EE was mainly composed of positive elements–“statements of loving attitudes”, “positive remarks”, while the negative dimensions—“self-sacrifice / overprotective behavior” and "emotional display"—which proved to be the most strongly related to EOI in the CFI—were observed in only 8.6% of the mothers.

Classically, over-involved behaviors are described as inappropriate behaviors [48]. Yet and in line with van Os et al. [49], maternal EOI may be an adaptive and necessary response, a supportive and kindly attitude towards the patient’s struggle with the illness contributing to a better prognosis in our population of adolescent girls with AN [15]. FMSS-EOI may have a positive meaning among parents of these adolescents, whose overall functioning is compromised by an acute life-threatening psychiatric illness, which can lead to death if they are not hospitalized [50–52]. Furthermore, more recent evidence suggests that some of the parental attitudes captured by EOI may be more developmentally appropriate in families with young children than in families who are dealing with an adult [15, 50–52]. For example, when parents of adolescents recount details about the birth or infancy of their teenager (“excessive details about the past”) this may be construed as appropriate and may not necessarily signal a problem in the relationship [15, 51]. But, there is little support to the adaptive role of Statements of (loving) Attitudes [15]. Maternal statements of attitude were found to be correlated with patients’ better clinical state in one study [18] while not in another one [19]. And paternal statements of attitudes were explained by any of the variables considered [19]. On a behavioral level, parents with a high EOI score have been described as attempting to soften events by placing themselves as a buffer between the patient and the outside world [48].

In other words the EOI dimension could indicate the degree to which the parent is involved (but not necessarily over-involved) in the care of their child in the course of the illness [15, 52–54].

In line with this positive interpretation of EE, our results are fairly similar to those from two studies conducted by Le Grange et al., using the S-CFI [26, 27] and reporting that parental warmth was significantly more frequent in the group of patients who had good outcome. Yet, parental warmth was found to have a complex relationship with relapse, and high ratings for warmth are often associated with high EOI levels in adults with schizophrenia [13, 55].

Beyond the absence of a link between paternal levels of EOI and outcome in our sample, it might be wise to look at dimensions of fathers’ psychological functioning which could mediate EE levels [18, 20, 21, 56–58]. Indeed, in a previous study, we observed that paternal EOI levels were not associated with any of his daughter’s clinical state but only with his own level of anxiety [18]. These results need to be enriched since our data were collected at discharge from the hospital [19].

Furthermore, our findings suggest that, first, maternal and paternal EE differentially influence outcome; and second, parental psychopathology and parental attributions are two distinct factors that influence parental EE levels [18–21, 48, 56]. In addition, not only parental and illness-related characteristics mediate EE, but the interaction between the two does as well [21, 22, 59]. These elements may be directly targeted in clinical settings. For this purpose, implementing psycho-educational and support groups (multiple-family and parents groups) may be helpful to families dealing with an adolescent suffering with AN. With this sort of ‘emotion coaching’- both parents and adolescent would be guided to address their emotions and help them find ways to express themselves in a healthy manner. It might encourage them to regulate their responses to life challenges. While, in the current context there is a tendency to reduce the gender differences, we have to keep in mind that studies support the fact that gender differences in positive emotion expressions are clearly observed by adolescence and adulthood [60]. Within this setting, it may also be easier to recognize and validate positive aspects of maternal EE. Regarding fathers, such groups may help them express their emotional experiences, including anxiety [18], and gain peer support. This approach may contribute to lowering paternal EE levels and booster their role as caregivers. Such recommandations may also be applied to single family therapy settings.

Our work shows certain limitations, such as sample size, considering that levels of parental EE were relatively low and the number of analyses was relatively large. We also did not register the exact number of hours the patients spent with their parents but all patients were submitted to the same condition. Recently, a few publications have started to mention this interesting variable (such as number of contact hours, AN subtype) [9, 10, 20–22, 61]; indeed, it should be reported systematically in future research. In addition, patients who underwent family therapy may have been more privileged with regard to coping with negative situations at home, although ED-related difficulties are targeted in both treatment groups by the psychiatrist. Nonetheless, it would be interesting to explore these predictive variables in the future [20, 21]. For reasons of statistical power, not all the EE sub-dimensions were represented. Our results are therefore exploratory and need confirmation in future studies.

EE has been studied as an aspect of family functioning that portends a poorer outcome for adolescents with AN [9, 10, 22, 46]. However, maternal emotional overinvolvement seems, in our study, to play a positive role in clinical outcome [20–21, 62]. Exploring the positive aspects of maternal EE could enhance our understanding of the role family interactions play in the course and treatment of AN.

## Supporting information

S1 Table **Supporting info summer2017_R1.docx**. (DOCX)
