# Supplementary material for: Predictive factors for outcome in adolescents with anorexia nervosa: To what extent does parental Expressed Emotion play a role?
Source: PLoS One. 2018 Jul 31;13(7):e0196820. doi: 10.1371/journal.pone.0196820 (PMC6067718; doi:10.1371/journal.pone.0196820)
Supplement: S1 Table — (DOCX) [file pone.0196820.s001.docx]

**Supporting information**

S1 Table 1 supplementary Material. Univariate regression models predictors of 18-month outcome criteria.

| **18-MONTH OUTCOME CRITERIA** | **FACTORS AT BASELINE PREDICTIVE OF OUTCOME** | **R^2^** | **p-value** | **unstandardized β** | **Related effect size OR [95% CI]** |
| --- | --- | --- | --- | --- | --- |
| **Good and intermediate MR outcome categories** | - Minimum lifetime BMI | .012 | .486 | -.194 | .824 [.0478; 1.420] |
|  | - Current BMI | .045 | .178 | -.410 | .664 [.0366; 1.205] |
|  | - Age an AN onset | .020 | .363 | .167 | 1.182 [.825; 1.694] |
|  | - AN duration | .009 | .540 | .027 | 1.028 [.942; 1.121] |
|  | - Duration of the index hospitalization | .000 | .952 | .001 | 1.001 [.961; 1.043] |
|  | - EDI | .001 | .884 | -.001 | .999 [.963; 1.015] |
|  | - Number of previous hospitalization | .015 | .448 | .245 | 1.277 [.679; 2.402] |
|  | - Maternal High Critical EE | .000 | .906 | .080 | 1.083 [.287; 4.095] |
|  | - Maternal Initial Statement (-) | .036 | .999 | -20.399 | .000 [.000; ] |
|  | - Maternal Relationship (-) | .072 | .999 | -20.453 | .000 [.000; ] |
|  | - Maternal Critical Comments (P) | .000 | .906 | .080 | 1.083 [.287; 4.095] |
|  | - Maternal Dissatisfaction (P) | .001 | .875 | .095 | 1.100 [.337; 3.590] |
|  | - Maternal High EOI EE | .001 | .875 | .095 | 1.100 [.337; 3.590] |
|  | - Maternal Emotional Display (P) | .054 | .999 | 20.425 | .000 [.000; ] |
|  | - Maternal Statement of (loving) Attitudes (P) | .001 | .843 | -.182 | .833 [.138; 5.047] |
|  | - Maternal Self-Sacrifice /Overprotective /Lack of Objectivity (P) | .036 | .999 | 20.399 | .000 [.000; ] |
|  | - Maternal Excessive Detail about the Past (P) | .140 | **.018** | -1.453 | .234 [.070; .778] |
|  | - Maternal Positive Remarks (P) | .006 | .610 | -.405 | .667 [.140; 3.172] |
|  | - Paternal High Critical EE | .001 | .856 | -.118 | .889 [.249; 3.168] |
|  | - Paternal Initial Statement (-) | .017 | .418 | .847 | 2.333 [.300; 18.146] |
|  | - Paternal Relationship (-) | .043 | .214 | 1.569 | 4.800 [.404; 57.025] |
|  | - Paternal Critical Comments (P) | .031 | .298 | .880 | 2.411 [.460; 12.622] |
|  | - Paternal Dissatisfaction (P) | .001 | .815 | -.140 | .870 [.269; 2.808] |
|  | - Paternal High EOI EE | .039 | .219 | -.742 | .476 [ .146; 1.557] |
|  | - Paternal Emotional Display (P) | .043 | .214 | -1.569 | .208 [.018; 2.475] |
|  | - Paternal Statement of (loving) Attitudes (P) | .054 | .153 | -.982 | .375 [.098; 1.438] |
|  | - Paternal Self-Sacrifice /Overprotective /Lack of Objectivity (P) | .020 | 1.000 | 20.453 | 762863120 [.000; ] |
|  | - Paternal Excessive Detail about the Past (P) | .016 | .434 | .492 | 1.636 [.477; 5.613] |
|  | - Paternal Positive Remarks (P) | .043 | .999 | 20.540 | 832214313 [.000; ] |
| **GOAS score** | - Minimum lifetime BMI | .012 | .417 | .221 | .108 [-.321; .764] |
|  | - Current BMI | .071 | **.041** | .596 | .267 [.025; 1.166] |
|  | - Age at AN onset | .006 | .572 | .103 | .075 [-.260; .465] |
|  | - AN duration | .000 | .874 | -.007 | -.021 [-.093; .079] |
|  | - Duration of the index hospitalization | .000 | .994 | .000 | -.001 [-.042; .042] |
|  | - EDI | ..017 | .320 | -.008 | -.132 [-.025; .008] |
|  | - Number of previous hospitalization | .062 | **.062** | -.559 | -.249 [-1.148; .030] |
|  | - Maternal High Critical EE | .034 | .172 | -.950 | -.184 [-2.324; .424] |
|  | - Maternal Initial Statement (-) | .007 | .547 | .984 | .081 [-2.275; 4.244] |
|  | - Maternal Relationship (-) | .027 | .221 | 1.434 | .164 [-.890; 3.757] |
|  | - Maternal Critical Comments (P) | .034 | .172 | -.950 | -.184 [-2.324; .424] |
|  | - Maternal Dissatisfaction (P) | .014 | .386 | .540 | .117 [-.698; 1.779] |
|  | - Maternal High EOI EE | .089 | **.024** | 1.379 | .299 [.188; 2.569] |
|  | - Maternal Emotional Display (P) | .020 | .295 | 1.407 | .141 [-1.260; 4.075] |
|  | - Maternal Statement of (loving) Attitudes (P) | .061 | *.063* | 1.798 | .248 [-.102; 3.697] |
|  | - Maternal Self-Sacrifice /Overprotective /Lack of Objectivity (P) | .000 | .899 | .207 | .017 [-3.062; 3.477] |
|  | - Maternal Excessive Detail about the Past (P) | .029 | .206 | .772 | .170 [-.436; 1.981] |
|  | - Maternal Positive Remarks (P) | .016 | .342 | .822 | .128 [-.896; 2.540] |
|  | - Paternal High Critical EE | .004 | .650 | .314 | .063 [-1.067; 1.696] |
|  | - Paternal Initial Statement (-) | .000 | .880 | .179 | .021 [-2.188; 2.545] |
|  | - Paternal Relationship (-) | .010 | .483 | -.948 | -.098 [-3.642; 1.745] |
|  | - Paternal Critical Comments (P) | .000 | .937 | .061 | .011 [-1.479; 1.600] |
|  | - Paternal Dissatisfaction (P) | .001 | .795 | -.165 | -.036 [-1.436; 1.106] |
|  | - Paternal High EOI EE | .034 | .180 | .864 | .185 [-.412; 2.140] |
|  | - Paternal Emotional Display (P) | .000 | .885 | .195 | .020 [-2.511; 2.902] |
|  | - Paternal Statement of (loving) Attitudes (P) | .001 | .843 | .147 | .028 [-1.331; 1.624] |
|  | - Paternal Self-Sacrifice /Overprotective /Lack of Objectivity (P) | .032 | .196 | 2.956 | .179 [-1.568; 7.481] |
|  | - Paternal Excessive Detail about the Past (P) | .010 | .473 | .460 | .100 [-.817; 1.737] |
|  | - Paternal Positive Remarks (P) | .000 | .970 | -.063 | -.005 [-3.405; 3.279] |
| **BMI** | - Minimum lifetime BMI | .011 | .419 | .214 | .106 [-.313; .742] |
|  | - Current BMI | .041 | .120 | .460 | .203 [-.123; 1.043] |
|  | - Age at AN onset | .003 | .682 | -.075 | -.054 [-.441; .290] |
|  | - AN duration | .003 | .693 | -.017 | -.052 [-.102; .068] |
|  | - Duration of the index hospitalization | .005 | .581 | .012 | .073 [-.030; .054] |
|  | - EDI | .012 | .408 | .007 | .109 [-.010; .024] |
|  | - Number of previous hospitalization | .004 | .649 | -.138 | -.062 [-.742; .466] |
|  | - Maternal High Critical EE | -0.006 | .422 | -.558 | -.108 [-1.938; .823] |
|  | - Maternal Initial Statement (-) | .033 | .171 | 2.217 | .182 [-.984; 5.419] |
|  | - Maternal Relationship (-) | .060 | *.063* | 2.152 | .246 [-.121; 4.424] |
|  | - Maternal Critical Comments (P) | .012 | .422 | -.558 | -.108 [-1.938; .823] |
|  | - Maternal Dissatisfaction (P) | .004 | .658 | -.275 | -.059 [-1.509; .959] |
|  | - Maternal High EOI EE | .005 | .599 | .325 | .070 [-.908; 1.558] |
|  | - Maternal Emotional Display (P) | .020 | .286 | -1.428 | -.142 [-4.083; 1.227] |
|  | - Maternal Statement of (loving) Attitudes (P) | .003 | .686 | .395 | .054 [-1.553; 2.343] |
|  | - Maternal Self-Sacrifice /Overprotective /Lack of Objectivity (P) | .001 | .863 | -.282 | -.023 [-3.538; 2.973] |
|  | - Maternal Excessive Detail about the Past (P) | .105 | **.013** | 1.460 | .324 [.319; 2.601] |
|  | - Maternal Positive Remarks (P) | .000 | .962 | .041 | .006 [-1.682; 1.764] |
|  | - Paternal High Critical EE | .000 | .913 | -.076 | -.015 [-1.542; 1.301] |
|  | - Paternal Initial Statement (-) | .006 | .580 | .654 | .076 [-1.700; 3.008] |
|  | - Paternal Relationship (-) | .002 | .759 | -.415 | -.309 [-3.112; 2.282] |
|  | - Paternal Critical Comments (P) | .002 | .767 | -.228 | -.041 [-1.759; 1.304] |
|  | - Paternal Dissatisfaction (P) | .001 | .804 | .157 | .034 [-1.104; 1.418] |
|  | - Paternal High EOI EE | .019 | .310 | .653 | .140 [-.624; 1.930] |
|  | - Paternal Emotional Display (P) | .002 | .337 | .453 | .046 [-2.243; 3.150] |
|  | - Paternal Statement of (loving) Attitudes (P) | .001 | .806 | .181 | .034 [-1.291; 1.653] |
|  | - Paternal Self-Sacrifice /Overprotective /Lack of Objectivity (P) | .000 | .907 | -.267 | -.016 [-4.855; 4.320] |
|  | - Paternal Excessive Detail about the Past (P) | .010 | .476 | .449 | .098 [-.807; 1.705] |
|  | - Paternal Positive Remarks (P) | .007 | .547 | -1.000 | -.085 [-4.314; 2.314] |
| **Menstruation** | - Minimum lifetime BMI | .016 | .410 | .206 | 1.229 [.753; 2.007] |
|  | - Current BMI | .027 | .290 | .288 | 1.334 [.782; 2.277] |
|  | - Age at AN onset | .015 | .876 | -.133 | .876 [.630; 1.217] |
|  | - AN duration | .000 | .956 | .002 | 1.002 [.929; 1.081] |
|  | - Duration of the index hospitalization | .032 | .256 | .203 | 1.023 [.983; 1.065] |
|  | - EDI | .109 | **.037** | .018 | 1.018 [1.001; 1.035] |
|  | - Number of previous hospitalization | .046 | .175 | -.389 | .678 [.386; 1.189] |
|  | - Maternal High Critical EE | .005 | .638 | -.301 | .740 [.212; 2.587] |
|  | - Maternal Initial Statement (-) | .000 | .957 | .077 | 1.80 [.064; 18.204] |
|  | - Maternal Relationship (-) | .007 | .603 | -.654 | .520 [.044; 6.101] |
|  | - Maternal Critical Comments (P) | .005 | .638 | -.301 | .740 [.212; 2.587] |
|  | - Maternal Dissatisfaction (P) | .027 | .293 | -.593 | .553 [.183; 1.670] |
|  | - Maternal High EOI EE | .000 | .933 | .048 | 1.049 [.343; 3.210] |
|  | - Maternal Emotional Display (P) | .011 | .519 | -.811 | .44 [.038; 5.216] |
|  | - Maternal Statement of (loving) Attitudes (P) | .008 | .581 | -.528 | .590 [.090; 3.846] |
|  | - Maternal Self-Sacrifice /Overprotective /Lack of Objectivity (P) | .000 | .957 | -.077 | .926 [.055; 15.607] |
|  | - Maternal Excessive Detail about the Past (P) | .009 | .555 | .327 | 1.387 [.469; 4.101] |
|  | - Maternal Positive Remarks (P) | .002 | .764 | .245 | 1.278 [.257; 6.345] |
|  | - Paternal High Critical EE | .002 | .796 | -.163 | .850 [.248; 2.911] |
|  | - Paternal Initial Statement (-) | .006 | .628 | -.610 | .543 [.046; 6.402] |
|  | - Paternal Relationship (-) | .067 | .999 | -21.162 | .000 [.000; ] |
|  | - Paternal Critical Comments (P) | .022 | .365 | -.651 | .522 [.128; 2.132] |
|  | - Paternal Dissatisfaction (P) | .010 | .540 | -.357 | .700 [.224; 2.188] |
|  | - Paternal High EOI EE | .003 | .756 | -.182 | .833 [.264; 2.633] |
|  | - Paternal Emotional Display (P) | 0.100 | .999 | 21.203 | 1.61E+9 [.000; ] |
|  | - Paternal Statement of (loving) Attitudes (P) | .104 | .101 | 1.778 | 5.918 [.705; 49.676] |
|  | - Paternal Self-Sacrifice /Overprotective /Lack of Objectivity (P) | .039 | 1.000 | -21.363 | .000 [.000; ] |
|  | - Paternal Excessive Detail about the Past (P) | .174 | **.011** | -1.589 | .204 [.606; .689] |
|  | - Paternal Positive Remarks (P) | .033 | 1.000 | 21.036 | 1.367E+9 [.000; ] |
| **EDI Score** | - Minimum lifetime BMI | .007 | .560 | -2.401 | -.081 [-10.608; 5.807] |
|  | - Current BMI | .073 | **.048** | -8.015 | -.271 [-15.949; -.080] |
|  | - Age at AN onset | .034 | .185 | 3.625 | .183 [-1.612; 8.141] |
|  | - AN duration | .001 | .835 | .121 | .029 [-1.036; 1.277] |
|  | - Duration of the index hospitalization | .077 | **.042** | .566 | .277 [.021; 1.111] |
|  | - EDI | .445 | **.000** | .553 | .667 [.381;.725] |
|  | - Number of previous hospitalization | .028 | .238 | 4.637 | .166 [-3.164; 12.438] |
|  | - Maternal High Critical EE | .007 | .558 | 5.700 | .083 [-13.716; 25.116] |
|  | - Maternal Initial Statement (-) | .003 | .705 | 11.333 | .054 [-48.353; 71.020] |
|  | - Maternal Relationship (-) | .013 | .424 | -14.027 | -.113 [-49.008; 20.954] |
|  | - Maternal Critical Comments (P) | .007 | .558 | 5.700 | .083 [-13.716; 25.116] |
|  | - Maternal Dissatisfaction (P) | .004 | .643 | -4.000 | -.066 [-21.218; 13.218] |
|  | - Maternal High EOI EE | .099 | **.023** | -18.842 | -.314 [-35.028; -2.657] |
|  | - Maternal Emotional Display (P) | .020 | .286 | -1.428 | -.142 [-4.083; 1.227] |
|  | - Maternal Statement of (loving) Attitudes (P) | .137 | **.007** | -33.478 | -.370 [-57.348; -9.608] |
|  | - Maternal Self-Sacrifice /Overprotective /Lack of Objectivity (P) | .009 | .506 | -14.160 | -.094 [-56.657; 28.337] |
|  | - Maternal Excessive Detail about the Past (P) | .000 | .936 | -.673 | -.012 [-17.287; 15.942] |
|  | - Maternal Positive Remarks (P) | .048 | .118 | -17.568 | -.219 [-39.766; 4.630] |
|  | - Paternal High Critical EE | .012 | .455 | -7.071 | -.109 [-25.974; 11.831] |
|  | - Paternal Initial Statement (-) | .021 | .318 | 15.572 | .146 [-15.468; 46.612] |
|  | - Paternal Relationship (-) | .041 | .163 | 24.732 | .203 [-10.358; 59.822] |
|  | - Paternal Critical Comments (P) | .000 | .948 | .690 | .009 [-20.624; 22.004] |
|  | - Paternal Dissatisfaction (P) | .000 | .973 | -.298 | -.005 [-17.656; 17.061] |
|  | - Paternal High EOI EE | .001 | .864 | 1.507 | .025 [-16.118; 19.133] |
|  | - Paternal Emotional Display (P) | .000 | .884 | 2.609 | .021 [-33.216; 38.433] |
|  | - Paternal Statement of (loving) Attitudes (P) | .004 | .679 | 4.070 | .061 [-15.563; 23.703] |
|  | - Paternal Self-Sacrifice /Overprotective /Lack of Objectivity (P) | .038 | .179 | -40.375 | -.195 [-99.965; 19.215] |
|  | - Paternal Excessive Detail about the Past (P) | .089 | **.038** | -18.318 | -.298 [-35.546; -1.090] |
|  | - Paternal Positive Remarks (P) | .063 | *.089* | 36-556 | .251 [-5.830; 78.941] |
| **Re-hospitalization for AN or other psychiatric disorder** | - Minimum lifetime BMI | .058 | .116 | -.395 | .673 [.411; 1.103] |
|  | - Current BMI | .292 | .001 | -1.216 | .296 [.143; .616] |
|  | - Age at AN onset | .020 | .363 | .167 | 1.182 [.825; 1.694] |
|  | - AN duration | .001 | .829 | .008 | 1.008 [.936; 1.086] |
|  | - Duration of the index hospitalization | .099 | **.047** | .044 | 1.045 [1.000; 1.091] |
|  | - EDI | .001 | .871 | .001 | 1.001 [.987; 1.016] |
|  | - Number of previous hospitalization | .822 | .996 | 21.610 | 2.428E+9 [.000; ] |
|  | - Maternal High Critical EE | .001 | .882 | .091 | 1.095 [.329; 3.648] |
|  | - Maternal Initial Statement (-) | .061 | .999 | 21.203 | 1.615E+9 [.000; ] |
|  | - Maternal Relationship (-) | .022 | .354 | 1.099 | 3.000 [.293; 30.686] |
|  | - Maternal Critical Comments (P) | .001 | .882 | .091 | 1.095 [.329; 3.648] |
|  | - Maternal Dissatisfaction (P) | .031 | .245 | -.648 | .523 [.175; 1.560] |
|  | - Maternal High EOI EE | .176 | **.007** | -1.660 | .190 [.057; .634] |
|  | - Maternal Emotional Display (P) | .007 | .600 | -.657 | .519 [.044; 6.057] |
|  | - Maternal Statement of (loving) Attitudes (P) | .014 | .446 | -.693 | .500 [.084; 2.972] |
|  | - Maternal Self-Sacrifice /Overprotective /Lack of Objectivity (P) | .061 | .999 | -21.203 | .000 [.000; ] |
|  | - Maternal Excessive Detail about the Past (P) | .075 | *.072* | .990 | 2.692 [.916; 7.909] |
|  | - Maternal Positive Remarks (P) | .010 | .514 | -.511 | .600 [.129; 2.785] |
|  | - Paternal High Critical EE | .006 | .619 | -.305 | .737 [.221; 2.459] |
|  | - Paternal Initial Statement (-) | .037 | .247 | -1.375 | .253 [.025; 2.599] |
|  | - Paternal Relationship (-) | .089 | .999 | 21.126 | 1.496E+9 [.000; ] |
|  | - Paternal Critical Comments (P) | .011 | .501 | -.469 | .626 [.160; 2.447] |
|  | - Paternal Dissatisfaction (P) | .016 | .419 | .452 | 1.571 [.526; 4.699] |
|  | - Paternal High EOI EE | .015 | .439 | .442 | 1.556 [.509; 4.758] |
|  | - Paternal Emotional Display (P) | .014 | .462 | .925 | 2.522 [.215; 29.577] |
|  | - Paternal Statement of (loving) Attitudes (P) | .038 | .233 | .846 | 2.331 [.581; 9.353] |
|  | - Paternal Self-Sacrifice /Overprotective /Lack of Objectivity (P) | .038 | 1.000 | 21.426 | 2.019E+9 [.000; ] |
|  | - Paternal Excessive Detail about the Past (P) | .018 | .390 | -.486 | .615 [.203; 1.864] |
|  | - Paternal Positive Remarks (P) | .000 | .891 | .197 | 1.217 [.072; 20.550] |

EE: Expressed Emotion; MR: Morgan and Russell; GOAS: Morgan and Russell Global Outcome Assessment Scale; EOI: Emotional Over-Involvement; (-): negative; (P): present; BMI: Body Mass Index; EDI: Eating Disorder Inventory; AN: Anorexia Nervosa; 95% CI: 95% Confidence Interval; OR= Odds Ratio; R^2^ Nagelkerke.
